# Monitoring for AF: Identifying the Burden of Atrial Fibrillation and Assessing Post-Ablation

**DOI:** 10.19102/icrm.2017.080104

**Published:** 2017-01-15

**Authors:** Rod S. Passman

**Affiliations:** ^1^Northwestern University Feinberg School of Medicine, Chicago, IL

**Keywords:** Arrhythmia, anticoagulation, atrial fibrillation, cardiac monitoring, implantable cardiac device

## Abstract

The management of atrial fibrillation (AF) is among the most challenging aspects of cardiology and uncertainties abound concerning stroke assessment and stroke risk reduction. Currently, AF is viewed as a dichotomous variable (fully present or absent) when it comes to stroke risk; there is no regard to the amount of AF either spontaneously or due to rhythm control strategies. For this reason, monitoring in patients with a known AF history, particularly after ablation, has focused on easily measured outcomes such as time to recurrence. However, emerging data suggest that thresholds exist between stroke risk and AF quantity as measured by either duration or burden. As a result, there is an increasing interest in long-term continuous monitoring following a rhythm control strategy to assess efficacy beyond typical symptom reduction. Insertable cardiac monitors (ICMs) with AF-sensing algorithms and remote data transmission capabilities can be used for this purpose, and wearable devices with similar functions are on the horizon. In addition to their diagnostic potential, these tools are also being used therapeutically with efforts to target anticoagulation therapy only in response to AF episodes.

## Introduction

Atrial fibrillation (AF) monitoring is among the most challenging aspects of arrhythmia management and is an area of intense scientific and clinical interest. The importance of monitoring patients with signs or symptoms of AF is self-evident. While the relationship between AF and stroke is well known, arrhythmia may also lead to heart failure, cognitive impairment, increased risk of hospitalization, and diminished lifespan.^1^ In addition, early AF detection can lead to interventions including stroke prevention measures, which are among the most successful treatments in cardiology. The major challenge lies in the fact that AF can be highly paroxysmal and completely asymptomatic, with some estimates showing that approximately 40% of patients are completely without symptoms. Stroke is the first manifestation of AF in at least 25% of all arrhythmia-related thromboembolic events. Unfortunately, symptoms are unreliable for the presence or extent of AF; most patients feel only a minority of their episodes, and even those with a known history of AF report related symptoms most often when they are in normal sinus rhythm.^2,3^ Beyond diagnosis, AF monitoring may have additional importance by providing information that can be used for stroke risk stratification and long-term decision making pertaining to anticoagulation issues. This review article will discuss the role of AF monitoring in determining AF duration and burden and their relationship with thromboembolic events. The role of AF monitoring post-ablation will also be discussed, as will available and future technologies for monitoring AF and the potential role of long-term monitoring to target specific AF therapies.

## Background

AF is the most common sustained arrhythmia in adults and affects 33.5 million patients worldwide.^4^ The triad of rate control, stroke prevention, and (in select individuals) rhythm control are the cornerstones of management. Guidelines attempt to summarize the best approaches to AF management, but it must be recognized that some widely practiced therapeutic principles are derived from studies performed before the advent of long-term cardiac monitoring, the development of which has raised important questions about several key aspects of AF.

The traditional view of AF defines the disease as a dichotomous entity that is entirely present or absent. This viewpoint originated with studies where the stroke risk was similar between those with permanent and non-permanent AF.^5^ As a result, guidelines do not distinguish between the “type” of AF (paroxysmal, persistent, or permanent) when it comes to assessing stroke risk or recommending stroke prevention strategies.^6^ However, it is worth noting that the landmark studies that serve as the basis for these recommendations relied on electrocardiogram documentation of AF detected on either routine exam or at the time of symptoms. The classification of AF as an “all or none” disease with a 30-s duration threshold needed for diagnosis has several major consequences. First, anticoagulation may be prescribed in individuals with brief, infrequent, or device-detected episodes where the risk of stroke may be lower than more overt presentations of AF. Second, those with a history of AF and other stroke risk factors are recommended to receive life-long anticoagulation even if AF recurrences are short-lived, infrequent, or altogether absent either spontaneously or due to a rhythm control strategy. Third, a threshold ≥ 30 s to define AF recurrence following a rhythm control intervention underestimates the success of such strategies and has no clinical relevance when it comes to stroke risk.

## AF duration, burden and density

The association between non-valvular AF and stroke has been known for decades, but there was little understanding of the relationship between the amount of AF and stroke risk until recently. The clinically practiced 48-h rule, allowing a patient with new-onset AF to be cardioverted to sinus rhythm without the need for transesophageal echocardiography or 3−4 weeks of therapeutic anticoagulation, suggests that there is a clear threshold between AF and thrombus formation.^6^ However, studies examining the relationship between AF duration and stroke questions whether even shorter AF durations can lead to thromboembolic events **(Table 1)**.^7–11^ Using long-term follow-up data from patients with dual chamber devices, these studies correlated thromboembolic risk with various AF duration thresholds. Collectively, these studies demonstrate that stroke risk increases with detected AF durations considerably shorter than the 48 h often used in clinical practice, yet the overall stroke risk from these studies is low, and no consistent threshold can be established for all individuals. It must also be remembered that AF is a progressive disease, and any decision to withhold anticoagulation based on a specific duration threshold would require ongoing monitoring to allow for prompt recognition when that threshold is crossed. It may be more accurate to view AF duration in conjunction with other risk factors rather than in isolation. One attempt to stratify stroke risk using a combination of AF duration and CHADS_2_ score demonstrated that stroke was associated with shorter AF durations in higher risk patients, suggesting that AF duration can be used to guide anticoagulation decisions in those with intermediate risk, while duration may not be relevant in those on the very low and higher ends of the spectrum.^12^ Ongoing studies such as NOAH (NCT02618577) and ARTESIA (NCT01938248) will better define the responsiveness of these shorter, device-detected “subclinical” AF episodes to anticoagulation.

While several studies have examined AF duration and stroke, the TRENDS study examined the relationship between stroke and AF burden.^13^ This prospective observational investigation of 2,486 patients with dual-chamber devices and one or more stroke risk factors defined atrial tachyarrhythmia (AT)/AF burden as the longest total duration on any given day during a 30-day rolling window before the first thromboembolic event or the end of the 1.4-year mean follow-up period. For those patients with any AT/AF, the median duration was 5.5 h. The annualized thromboembolic risk, including transient ischemic attacks (TIAs), was 1.1% for no AT/AF, 1.1% for “low” burden AT/AF (i.e. <5.5 h), and 2.4% for “high” burden (i.e. >5.5 h) subsets of the 30-day windows. While “high” burden AF conferred a doubling of thromboembolic risk, it must be recognized that nearly half the events in TRENDS were TIAs, and even those with AF burden ≥ 5.5 h had an annualized stroke risk similar to that of lower risk patients based on current risk scores.

**Figure 1:**
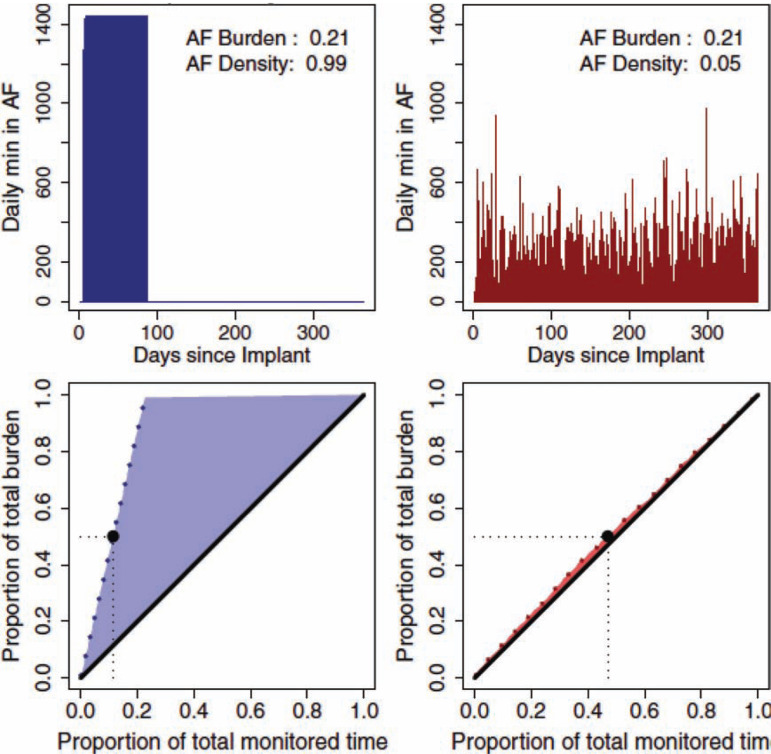
Reconstruction of AF burden and density in two patients with dual-chamber cardiac rhythm management devices.^14^ Two patients with the same AF burden but different AF densities. The patients on the left and right have high- and low-density AF, respectively. With the rhythm history reconstructed, the course of the minimum monitored time required for each burden proportion is plotted against the proportion of the total burden (dotted line, bottom). The left patient developed 50% of the total burden in 11% of the monitoring period (black dot, bottom left). In contrast, the right patient required 40% of the observation time to develop 50% of their AF burden (black dot, bottom right) as each day contributes less to the total burden because the AF burden is spread out over more days. The black diagonal lines on the bottom figures represent a hypothetical uniform AF burden distribution. The area between the actual (blue or red dotted lines) and uniform hypothetical (solid black diagonal) AF burden development is evaluated as a measure of the temporal aggregation of the AF burden (AF burden density). Abbreviation: AF: atrial fibrillation. Reprinted with permission from: Charitos EI, Stierle U, Ziegler PD, Baldewig M, Robinson DR, Sievers HH, Hanke T. A comprehensive evaluation of rhythm monitoring strategies for the detection of atrial fibrillation recurrence: insights from 647 continuously monitored patients and implications for monitoring after therapeutic interventions. *Circulation.* 2012;126(7):806−814. http://circ.ahajournals.org/content/126/7/806.

The concepts of AF duration and AF burden are conceptually easy but fail to address the temporal distribution of AF episodes over time. AF burden density is a dimensionless quantity that assumes values between 0 and 1. Values close to 0 indicate a low burden aggregation (i.e. AF episodes spread evenly throughout the monitored period), while values closer to 1 denote maximum burden as a temporal aggregation (a block of AF or the complete burden as one continuous episode.)^14^ **Figure 1** contains a reconstruction of the rhythm history of two patients both with identical AF burdens but with markedly disparate AF densities. To date, no studies have attempted to correlate AF density and stroke risk. However, AF density has major implications for AF detection; low AF densities are more easily identified by typical short-term external monitors, while high-density AF is considerably more elusive. This may be particularly relevant following AF ablation, as the majority of procedures are performed on patients with paroxysmal AF for whom recurrences are likely to be of high density and therefore unlikely to be detected by routine external monitoring techniques.

## Post-AF ablation monitoring

AF ablation is used with increasing frequency for the maintenance of normal sinus rhythm, particularly in those with symptomatic AF who have failed one or more antiarrhythmic drugs. While symptoms are the major indication for rhythm control, it is recognized that ablation reduces both AF burden and the ratio between symptomatic and asymptomatic AF episodes.^15^ Thus, symptom relief is an unreliable measure of AF ablation efficacy, so monitoring for recurrent AF following ablation is important on several levels.^16^ On an individual basis, monitoring may be important to correlate symptoms with AF recurrences and survey for asymptomatic episodes. From a scientific perspective, it is imperative to understand the success rates of various ablation techniques to establish treatment effects of approved therapies and compare them to new technologies and approaches. To date, there is no clear consensus on the most appropriate clinical endpoints for studies of rhythm management in AF, including ablation. Outcome measurements such as stroke and mortality have been used, but because these are typically low incident events, trial size and the long follow-up times required are major limitations. For these reasons, endpoints related to recurrence of AF have been favored. Time to recurrence is easily measured but has unproven clinical significance. It can be recorded by short-term external monitors worn for days or weeks or during symptoms. Unfortunately, time to recurrence correlates poorly with AF burden and underestimates the impact of ablation in individuals with drastic burden reductions who still have brief, somewhat infrequent episodes of AF on external monitoring.^17^ In contrast to time to recurrence, reductions in AF duration or burden may provide meaningful information, particularly if an AF-duration threshold for stroke is ultimately established by ongoing trials. The accurate measurement of AF duration or burden requires long-term cardiac monitoring, the options of which are discussed next.

## AF monitoring and management using implantable and wearable devices

**Figure 2:**
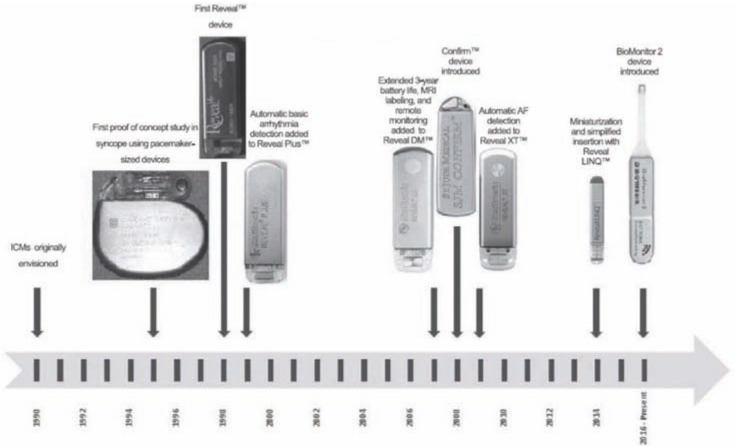
FDA-approved implantable/insertable cardiac monitors.^20^ Reprinted with permission from: Tomson TT, Passman R. Current and emerging uses of insertable cardiac monitors: evaluation of syncope and monitoring for atrial fibrillation. *Cardiol Rev.* 2017 Jan/Feb;25(1):22−29. http://insights.ovid.com/crossref?an=00045415-201701000-00007.

**Figure 3:**
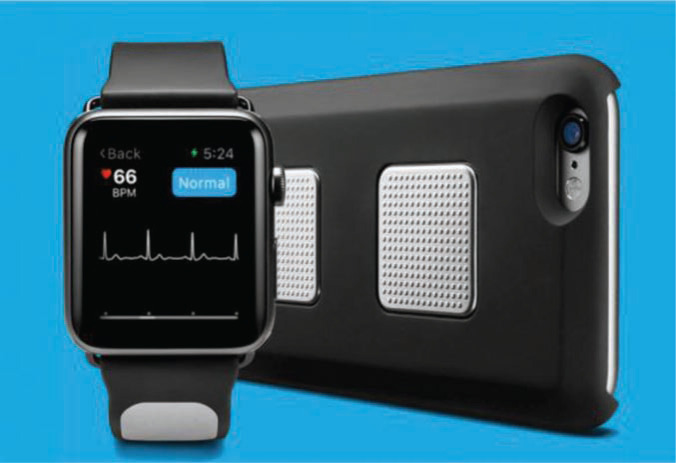
An AF-sensing smartwatch (not FDA approved) and smartphone adaptor (FDA approved).

Pacemakers and implantable cardioverter devices (ICDs) with atrial leads can detect local atrial depolarizations at the site contact with the myocardium and detect atrial arrhythmias with high sensitivity and specificity. Atrial rates above a certain cut off (typically 170−220 beats/min) can be categorized as atrial high rate episodes (AHREs). These AHREs can be triggered by any of the ATs including AF, atrial flutter, or atrial tachycardia, with AF and atrial flutter representing the most common arrhythmias.^18,19^ As the majority of AF patients have no indications for pacemakers or ICDs, insertable cardiac monitors (ICMs) have been developed that can detect AF based partly on irregularities in the R-R interval on far-field electrocardiogram **(Figure 2)**. These devices, initially developed for long-term monitoring of patients with unexplained syncope, can be placed subcutaneously over the left chest using local anesthetic in several minutes and are highly accurate for detecting AF.^20^ In addition, these devices can now be remotely monitored on a daily basis, providing an alert when a preprogrammed threshold event has occurred. As AF-detection algorithms are based primarily on R-R interval irregularities, false positives due to premature beats are not uncommon, particularly with short episodes.^21^ Furthermore, the devices are insensitive to AF episodes <2 min. However, ICM use in post-ablation patients is increasing both for research and clinical care indications and offers an attractive alternative to standard external monitoring, particularly if the information is necessary for clinical decision making. Still, the cost and invasive nature of these devices limits their widespread adoption and has fueled the development of wearable devices for similar purposes. Smartphones using photoplethysmyography or ultrasound adaptors are commercially available and have been used for community screening purposes.^22^ Necklaces, watches, and patches are all being developed with AF-sensing technologies that will allow assessment of AF duration, burden, and density well beyond the “snapshots” available with traditional monitors **(Figure 3)**.

**Figure 4:**
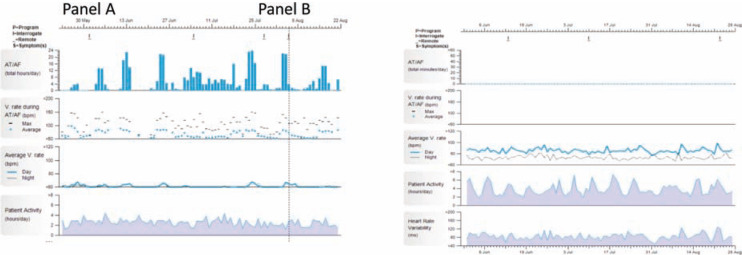
ICM findings from two patients with AF and CHA_2_DS_2_VASC scores of 3. These panels show the results of three-to-four months of continuous monitoring with ICMs in two patients with a history of AF. a: ICM tracings from a 68-year-old female with a history of hypertension (CHA_2_DS_2_VASC score = 3). The patient had numerous AF episodes lasting up to several days in duration (blue columns, top row). b: ICM tracings from a 68-year-old female with a history of hypertension (CHA_2_DS_2_VASC score = 3). The patient underwent AF ablation and had no AF over the monitoring period. Under current standards of care, both patients would be recommended to receive chronic oral anticoagulation. Abbreviations: AF: atrial fibrillation; CHA_2_DS_2_VASC: congestive heart failure, hypertension, age ≥75, diabetes mellitus, prior stroke or transient ischemic attack, vascular disease, age 65−74 years, sex; ICM: implantable cardiac monitor.

**Figure 5:**
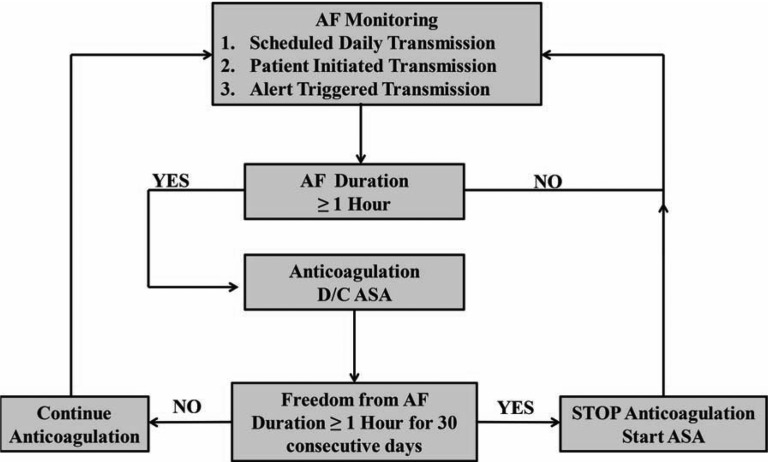
Study schema for the REACT.COM study.^26^ Reprinted with permission from: Passman R, Leong-Sit P, Andrei AC, Huskin A, Tomson TT, Bernstein, et al. Targeted anticoagulation for atrial fibrillation guided by continuous rhythm assessment with an insertable cardiac monitor: The rhythm evaluation for anticoagulation with continuous monitoring (REACT.COM) pilot study. *J Cardiovasc Electrophysiol.* 2016 Mar;27(3):264−270. http://onlinelibrary.wiley.com/doi/10.1111/jce.12864/abstract.

Current practice recommends life-long anticoagulation in patients with AF and stroke risk factors, regardless of whether rhythm control has been achieved with antiarrhythmic drugs or ablation.^6^ These recommendations are based on the findings of studies like AFFIRM where higher stroke risk was noted for patients who had their anticoagulation stopped during a rhythm control strategy.^23^ Unfortunately, this approach may expose some patients to the hemorrhagic risk of anticoagulation during long periods of sinus rhythm where the risk of stroke may be low **(Figure 4)**. Concerns about hemorrhagic events is one major reason why many patients choose to stop anticoagulation following ablation despite guideline recommendations and conflicting data on the safety of this decision.^24,25^ Instead of viewing anticoagulation as a life-long therapy for all patients with an AF history, the use of implantable or wearable long-term AF monitors may provide an opportunity for targeted anticoagulation only in response to an AF episode. In the REACT.COM pilot study, patients used an ICM for daily remote AF monitoring **(Figure 5)**.^26^ Using rapid-onset novel oral anticoagulants, patients only reinitiated anticoagulation following prolonged AF episodes. This cost-effective approach reduced the “time on” anticoagulation by 94% with no observed strokes.^26,27^ Further research will be needed to determine whether such an approach is as effective as chronic anticoagulation in terms of stroke risk and whether wearable devices that require no medical supervision can be used for a similar purpose but at a lower cost.

Several knowledge gaps need to be addressed to facilitate targeted anticoagulation. Some studies of patients with dual-chamber devices have demonstrated a temporal dissociation between AF and stroke, though the number of events has been small, and many strokes occurred in individuals with several potential mechanisms.^28^ In contrast, the largest study reported a 5-fold elevation in stroke risk within 30 days of an AF episode, demonstrating a clear temporal association between AF and stroke.^9^ Other evidence suggests that AF may be a marker for an “atriopathy” consisting of left atrial endothelial dysfunction, inflammation, platelet aggregation, and a local hypercoagulable state that may persist even in the absence of AF.^30,31^ As with most of medicine, all these findings may be true in some but not all individuals with AF. Long-term monitoring of a large AF population will provide critical insights into the relationship between AF and stroke and either fuel or end the debate between those who believe that AF is the cause of stroke or simply a marker for stroke risk even in the absence of an arrhythmia.

## Conclusions

This review highlights the methods and measures of AF monitoring and underscores the importance of viewing AF not as an “all or none” phenomenon but as an arrhythmia for which duration and burden carry clinical value. The use of long-term continuous monitoring may expand as we move to define the success of a rhythm control strategy beyond symptom relief and time to AF recurrence. Long-term monitoring, whether implantable or wearable, may provide an opportunity to target anticoagulation therapy only around the time of an AF episode, personalizing this challenging treatment aspect.

## Figures and Tables

**Table 1:** AF Duration and Stroke Risk in Five Main Trials^7–11^

Author (year)	Pts (n)	Study Type/Inclusion Criteria	Monitoring Method/Duration	Outcome
Glotzer et al. (2003)^7^	312	Ancillary analysis of multicenter RCT (MOST).	Dual-chamber PPM for a median of 27 months.	10 patients (32%) developed stroke. Atrial arrhythmias >5 min; HR 2.8, p = 0.0011 for death and non-fatal stroke.
Capucci et al. (2005)^8^	725	Prospective, registry study.	Dual chamber PPM for a median of 22 months.	14 patients (1.9%) had an arterial thromboembolic event. AF episode lasting >24 h: adjusted HR 3.1, p = 0.044 for embolic events. AF episodes >5 min: no difference in embolic events.
Healey et al. (2012)^9^	2580	Primary analysis of a multicenter RCT (ASSERT).	Dual chamber PPM or ICD for a mean of 2.5 yrs.	AT >6 min: HR 1.76, p = 0.05 for stroke or systemic embolism compared to patients with no arrhythmia. AT <17.7 h: annual rate of stroke or systemic embolism 1.2%. AT >17.7 h: annual rate of stroke or systemic embolism 4.9%.
Shanmugam et al. (2012)^10^	560	Ancillary analysis from two prospective multicenter observational studies of CHF patients with CRT.	CRT device for a mean of 1 year.	11 patients (2%) had a thromboembolic event. AT >3.8 hours a day; HR 9.4; p = 0.008 for stroke or systemic embolism compared to patients with no arrhythmia. No significant increase risk of thromboembolism events in patients with >3.8 h/d versus <3.8 h/d; HR 2.4; p = 0.23.
Swiryn et al. (2016)^11^	5379	Prospective, registry study (RATE).	Dual chamber PPM or ICD for a median of 22.9 months.	53 patients (0.99%) had stroke or TIA. No association between "short" or "long" episodes of AT/AF and thromboembolic events.

Abbreviations: AF: atrial fibrillation; AT: atrial tachyarrhythmia; CHF: congestive heart failure; CRT: cardiac resynchronization therapy; HR: hazard ratio; ICD: implantable cardioverter device; PPM: permanent implantable pacemaker; RCT: randomized controlled trial; TIA: transient ischemic attack.
